# Comprehensive Search for Genes Involved in Thalidomide Teratogenicity Using Early Differentiation Models of Human Induced Pluripotent Stem Cells: Potential Applications in Reproductive and Developmental Toxicity Testing

**DOI:** 10.3390/cells14030215

**Published:** 2025-02-02

**Authors:** Yu Kato, Takeshi Inaba, Koudai Shinke, Noriko Hiramatsu, Tetsuhiro Horie, Takuya Sakamoto, Yuko Hata, Eiji Sugihara, Tetsuya Takimoto, Noriaki Nagai, Yasuhito Ishigaki, Hajime Kojima, Osamu Nagano, Naoki Yamamoto, Hideyuki Saya

**Affiliations:** 1 Oncology Innovation Center, Research Promotion Headquarters, Fujita Health University, Toyoake 470-1192, Aichi, Japan; yu.toki@fujita-hu.ac.jp (Y.K.); eiji.sugihara@fujita-hu.ac.jp (E.S.); tetsuya.takimoto@fujita-hu.ac.jp (T.T.); osamu.nagano@fujita-hu.ac.jp (O.N.); hsaya@fujita-hu.ac.jp (H.S.); 2 Center for Society-Academia Collaboration, Research Promotion Headquarters, Fujita Health University, Toyoake 470-1192, Aichi, Japan; norikoh@fujita-hu.ac.jp; 3 Graduate School of Health Sciences, Fujita Health University, Toyoake 470-1192, Aichi, Japan; 82023103@fujita-hu.ac.jp (T.I.); 82023112@fujita-hu.ac.jp (K.S.); 4 Clinical Laboratory, Fujita Health University Hospital, Toyoake 470-1192, Aichi, Japan; 5 Medical Research Institute, Kanazawa Medical University, Uchinada 920-0293, Ishikawa, Japan; horie-te@kanazawa-med.ac.jp (T.H.); taku0731@kanazawa-med.ac.jp (T.S.); ishigaki@kanazawa-med.ac.jp (Y.I.); 6 Department of Pharmacy, Kanazawa Medical University Hospital, Uchinada 920-0293, Ishikawa, Japan; 7 Open Facility Center, Research Promotion Headquarters, Fujita Health University, Toyoake 470-1192, Aichi, Japan; yuko.hata@fujita-hu.ac.jp; 8 Faculty of Pharmacy, Kindai University, Higashiosaka 577-8502, Osaka, Japan; nagai_n@phar.kindai.ac.jp; 9 Department of Pharmaceutical Engineering, Faculty of Engineering, Sanyo-Onoda City University, Sanyo-Onoda 756-0884, Yamaguchi, Japan; h-kojima@rs.socu.ac.jp; 10 National Institute of Health Sciences (NIHS), Kawasaki 210-9501, Kanagawa, Japan; 11 International Center for Cell and Gene Therapy, Research Promotion Headquarters, Fujita Health University, Toyoake 470-1192, Aichi, Japan

**Keywords:** developmental toxicity, iPSCs, valproic acid, thalidomide, RNA-seq analysis, candidate genes, ICH S5(R3), alternative testing methods

## Abstract

Developmental toxicity testing is essential to identify substances that may harm embryonic development. This study aimed to establish a protocol for evaluating developmental toxicity using human induced pluripotent stem cells (iPSCs) by analyzing cellular activity and gene expression changes. Two ICH S5(R3) positive substances, valproic acid (VPA), which is a substance previously detected as positive by other test methods, and thalidomide (Thalido), were examined during early trichoderm differentiation without fetal bovine serum. RNA-seq analysis identified seven candidate genes, including *TP63*, associated with altered expression following exposure to VPA or Thalido. These genes were implicated in pathways related to tissue development, cell growth, and molecular interactions. While the assay effectively detected VPA and Thalido, its limitations include testing only soluble substances and focusing on early differentiation stages. Nevertheless, the protocol demonstrates potential for the classification and evaluation of emerging modality drugs based on physical properties such as solubility, polarity, and pH. Integration with AI analysis may enhance its capacity to uncover genetic variations and evaluate previously uncharacterized substances. This study provides a foundation for alternative developmental toxicity testing methods, with further refinements in the culture method expected to improve accuracy and applicability in regulatory toxicology.

## 1. Introduction

The worldwide adoption of the 3R (replacement, reduction, refinement) principles in animal testing has led to increasing use of alternative methods in safety evaluations for pharmaceuticals, cosmetics, and other products. In the pharmaceutical field, the International Council for Harmonisation of Technical Requirements for Pharmaceuticals for Human Use (ICH) guidelines already recommend in vitro and in silico methods for genotoxicity and photosafety studies. The use of alternative methods was explicitly incorporated into the ICH harmonized guideline [ICH-S5(R3)] for detecting reproductive and developmental toxicity in human pharmaceuticals in February 2020 [1]. However, the guideline does not specify which alternative methods, and its implementation is limited by insufficient quality and quantity of data. The purpose of reproductive and developmental toxicity testing using alternative methods is to assess and verify the safety of a drug or other substances for human reproduction and fetal development. This involves examining the effects of their application or administration on reproduction and fetal development in the maternal body.

Around 1960, teratogenicity (also known as “Thalidomide Embryopathy”) was reported in pregnant women who had taken thalidomide as a sleeping pill or to alleviate morning sickness during early pregnancy. The drug caused malformations in the limbs and internal organs of fetuses. Although not found to be developmentally toxic in rodents [2], thalidomide was found to be developmentally toxic in humans, monkeys, and rabbits, with rodents such as mice and rats found less sensitive to developmental toxicity [3].

When verifying the reproductive developmental toxicity test with cells, the initial differentiation stage from embryonic stem (ES) or induced pluripotent stem (iPS) cells as pluripotent stem cells, to the trichoderm is the first period to be addressed. The trichoderm consists of three cell layers (ectoderm, mesoderm, and endoderm) that form during the early stages of differentiation, with each layer giving rise to different organs and tissues. This process establishes the basic structures and functions of the body. The Embryonic Stem Cell Test (EST) method was developed as an in vitro assay using embryonic stem (ES) cells and whole embryo culture tests [4]. A simplified and higher-throughput version of this method, the Hand1-EST method, uses Hand1 as an indicator [5,6,7,8,9]. The Hand1 gene, which encodes a transcription factor, plays a role in regulating heart development and structure [10]. However, the Hand1-EST assay failed to identify thalidomide, known to cause developmental damage (teratogenicity) in human fetuses, as a positive substance for reproductive and developmental toxicity. Although several assays for reproductive and developmental toxicity have been developed, challenges remain. Despite its teratogenic effects, thalidomide has immunomodulatory and antitumor properties and is still used today to treat multiple myeloma and erythema nodosum leprosum.

This study aims to establish a testing system using human-derived cells to detect thalidomide and to develop a method capable of evaluating new drugs and other substances that may pose similar developmental risks (teratogenicity) in the future.

## 2. Materials and Methods

### 2.1. Verification of Used iPS Cells

The iPS cells used in this study were human vascular endothelial cell-derived iPS cells (RPChiPS 771-2, REPROCELL Inc., Kawasaki, Japan), generated using an RNA reprogramming kit (StemRNA Reprogramming kit, REPROCELL Inc., Telangana, India). Cell culture dishes (Eppendorf Corporate, Hamburg, Germany) were coated with iMatrix-511 (Matrixome Inc., Osaka, Japan). Cells were cultured in StemFit^®^ medium (Ajinomoto Healthy Supply Co., Inc., Tokyo, Japan) and incubated at 37 °C with 5%CO_2_. During passages, CultureSure^®^ 10 mmol/L Y-27632 solution (Fujifilm Wako Pure Chemicals Corp., Osaka, Japan) was added at a ratio of 1:1000 to the medium. The medium was changed daily, and passages were performed every 4–5 days. Mycoplasma testing was conducted using the e-Myco™ Mycoplasma PCR Detection Kit (iNtRON Biotechnology, Inc., Bucheon, Republic of Korea) to ensure the cells were infection free.

iPS cell evaluation was performed through alkaline phosphatase staining and immunostaining for markers, including SOX2, SSEA4, OCT3/4, TRA-1-60, and NANOG [11,12]. Immunostaining results were analyzed using a fluorescence microscope (Power IX-71 and DP-71; Olympus, Tokyo, Japan).

### 2.2. Differentiation Medium, Exposure Substances, and Schedule

Generally, iPS cells were differentiated using fetal bovine serum (FBS); however, variability among FBS lots may affect the outcomes of cell differentiation. To address this, medium conditions were optimized to replicate the differentiation observed with FBS (Supplementary Figure S1). The differentiation medium included the following components: 5% KnockOut™ Serum Replacement (KSR; Thermo Fisher Scientific, Inc., Waltham, MA, USA), 1% MEM Non-Essential Amino Acids Solution (MEM NEAA; Thermo Fisher Scientific Inc.), 2% GlutaMAX™ Supplement (GlutaMAX; Thermo Fisher Scientific Inc.), 1% Insulin-Transferrin-Selenium (ITS-X; Thermo Fisher Scientific Inc.), 1% StemSure^®^ 50 mmol/L Monothioglycerol Solution (Fujifilm Wako Pure Chemicals Corp.), 1% Penicillin (10,000 units/mL) and Streptomycin (10,000 µg/mL) solution (PC/SM; Fujifilm Wako Pure Chemicals Corp.), and Dulbecco’s Modified Eagle’s Medium (DMEM) at 4.5 g/L D-Glucose (Thermo Fisher Scientific Inc.).

CultureSure^®^ Dimethyl Sulfoxide (DMSO; Fujifilm Wako Pure Chemicals Corp.) was used as the solvent for dissolving and diluting the test substances. Comparisons were performed by adding 0.2% (*w*/*v*) or 0.1% (*w*/*v*) of the DMSO stock solution to the differentiation medium, and the results were almost identical (Supplementary Figure S2). Thus, the test substance stock solutions were diluted in differentiation medium containing 0.2% (*w*/*v*) DMSO, which was also used as the control medium.

Items listed in ICH S5(R3) were used as test substances (Supplementary Table S1) [1]. Negative controls included Saxagliptin (Saxa, Cas No. 361442-04-8; BioVision, Milpitas, CA, USA) and Vildagliptin (Vilda, Cas No. 274901-16-5; Sigma Aldrich Co. LLC, St. Louis, MO, USA). Valproic Acid (VPA, Cas No. 99-66-1; Fujifilm Wako Pure Chemicals Corp.), included as a positive control, has been detected in other reproductive developmental toxicity studies. Thalidomide (Thalido, Cas No. 50-35-1; Tokyo Chemical Industry Co., Ltd., Tokyo, Japan), included as an ICH S5(R3) positive substance was also tested. Solid test substances were weighed using an electronic balance (HR-251A; A&D Company, Tokyo, Japan). Since VPA is a liquid, it was diluted with DMSO to prepare the test stock solution.

To confirm the differentiation of iPS cells into early trichoblast-like cells, the cells were cultured for 6 days in differentiation medium, based on a previously reported developmental toxicity study using mouse ES cells [5,6,7,8,9]. Differentiated iPS cells served as the assay reference, and the assay was performed using the TaqMan™ hPSC Scorecard™ Panel (Thermo Fisher Scientific) following the manufacturer’s instructions. A QuantStudio 7 real-time PCR system (Thermo Fisher Scientific) was used for the assay.

**Figure 1:**
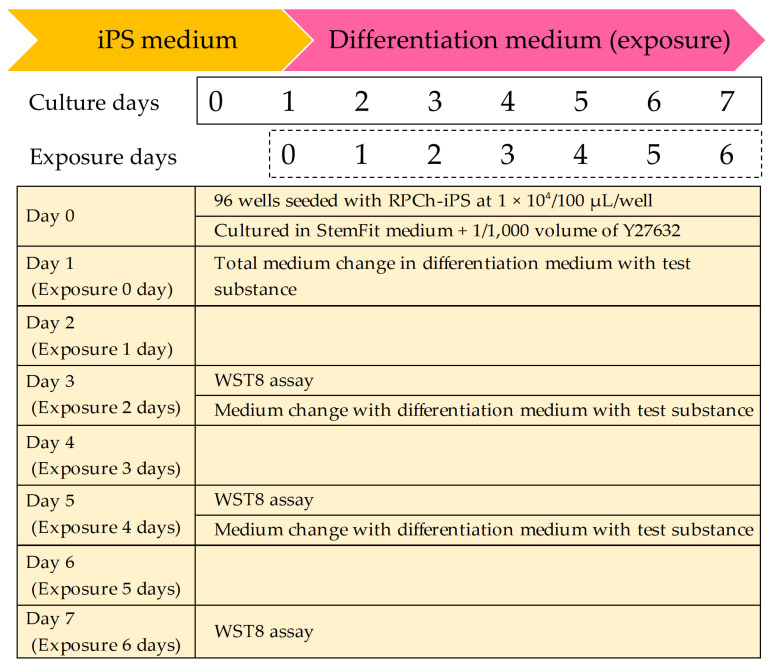
Schedule of 6 days of continuous exposure to test substances using differentiation media.

iPS cells were seeded onto iMatrix-511-coated BioLite™ 96-well plates (Thermo Fisher Scientific, Cat. No. 130188) at a density of 1 × 104 cells/well. Cell viability was assessed on days 2, 4, and 6 of exposure using a Multiskan™ FC absorbance microplate reader (Thermo Fisher Scientific, Cat No. 130188) and Cell Counting Kit-8 (WST-8; Dojindo Laboratories Co., Mashiki, Japan). Absorbance was measured at a primary wavelength of 450 nm, and a reference wavelength of 650 nm. IPS cells cultured in differentiation medium with 0.2% (*w*/*v*) DMSO served as the control. (Figure 1).

The maximum applicable concentration of each test substance was determined based on its cytotoxicity and solubility in the culture medium without forming crystals or precipitates after 1 day of exposure. The cytotoxicity threshold was defined as the concentration at which cell viability, measured by WST-8 assay, was ≥90% the control after 6 days of exposure. Conversely, the lowest applicable concentration was set as the highest blood concentration (Cmax) estimated from the test substance medical product information. Criteria to determine the concentration range of each test substance is shown in Table 1.

### 2.3. Gene Expression Analysis Using RNA-Sequencing (RNA-Seq)

Total RNA was extracted from cells on days 1 and 6 of exposure to test substances or the control (differentiation medium without test substances) using the RNeasy^®^ Mini Kit (QIAGEN N.V., Limburg, The Nederlands). Library preparation was conducted using the NEBNext Poly(A) mRNA Magnetic Isolation Module and the NEBNext Ultra II Directional RNA Library Prep Kit for Illumina (New England Biolabs Inc., Biolabs Inc., Ipswich, MA, USA). Sample quality was verified with an Agilent TapeStation system (Agilent Technologies, Inc., Santa Clara, CA, USA), and sample quantification was performed with a Quantus Fluorometer (Promega Corp., Madison, WI, USA). Multiplexed library pools were sequenced using 50 bp paired-end reads on a NextSeq2000 platform (Illumina, Inc., San Diego, CA, USA) with NextSeq 1000/2000 P1 and P2 Reagents (v3). Sequencing reads were trimmed and aligned to the human reference human genome “GRCh38”. The Sequence Read Archive accession number for the RNA-Seq sequence data were SUB14990084. The corresponding BioSample accession numbers were as follows: SAMN46116148, SAMN46116149, SAMN46116150, SAMN46116151, SAMN46116152, SAMN46116153, SAMN46116154, SAMN 46116155, SAMN46116156, and SAMN46116157. RNA-seq data processing was performed using the CLC Genomics Workbench (ver22.0.5; QIAGEN). Heatmap analysis was conducted using TPM (Transcripts per Million) values. Gene ontology (GO) analysis was performed with the DAVID Bioinformatics tool (National Institutes of Health, USA, v2023q4). The Benjamini–Hochberg method was used to calculate the FDR (False Discovery Rate) for statistical significance.

### 2.4. Gene Expression Reanalysis Using Quantitative Real-Time Polymerase Chain Reaction (qPCR)

Total RNA was extracted from cells on days 1 and 6 of exposure to differentiation medium with or without test substances (control) using the TaqMan™ Gene Expression Cells-to-CT™ kit (Thermo Fisher Scientific). Reverse transcription was performed to generate complementary DNA (cDNA). Each cDNA sample was mixed with TaqMan™ Gene Expression Master Mix (Thermo Fisher Scientific) and TaqMan™ Gene Expression Assays (TaqMan Primer & Probe, Thermo Fisher Scientific), then subjected to qPCR using a real-time PCR system (Quant Studio 1, Thermo Fisher Scientific). The TaqMan™ Primer & Probe sets used for qPCR are listed in Table 2. Actin beta was used as the endogenous control, and comparative quantitative analysis was performed using the ΔΔCt method relative to the control.

### 2.5. Statistical Analyses

One-way analysis of variance or the Kruskal–Wallis test was performed using the Statistical Package for Social Science Statistics software version 24 (IBM, New York, NY, USA) with *p* < 0.05 deemed statistically significant. Results were expressed as means ± SD. GO analysis was conducted using DAVID Bioinformatics. FDRs were calculated using the Benjamini–Hochberg method.

## 3. Results

### 3.1. Validation of iPS Cells

**Figure 2:**
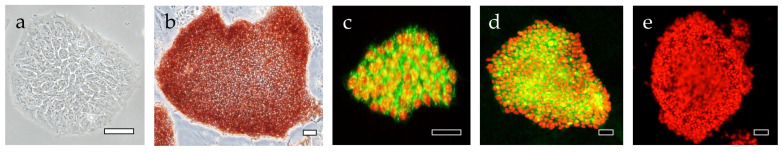
Morphology and staining results of iPS cells. (**a**) Inverted micrograph of an iPS cell colony. (**b**) Alkaline phosphatase staining. (**c**) Immunofluorescent antibody staining for SOX2 (red) and SSEA4 (green). (**d**) Immunofluorescent antibody staining for OCT3/4 (red) and TRA-1-60 (green). (**e**) Immunofluorescent antibody staining for NANOG (red). Scale bar: 50 µm.

The iPS cells used in this study formed colonies (Figure 2a) and were positive for alkaline phosphatase staining (Figure 2b). Immunostaining confirmed positivity for SOX2 and SSEA-4 (Figure 2c), OCT3/4 and TRA-1-60 (Figure 2d), and NANOG (Figure 2e). Mycoplasma PCR tests for the iPS cells were negative.

### 3.2. Differentiation of iPS Cells into Early Trichoderm Using Differentiation Medium

**Figure 3:**
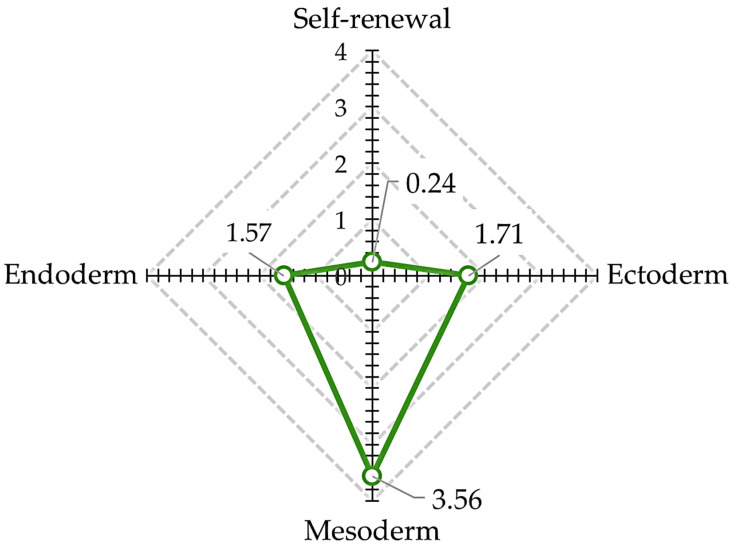
Comparative analysis of trichoderm gene expression after differentiation induction. After 6 days of culture in differentiation induction medium, the gene expression levels of germ layer-specific markers increased compared to those of undifferentiated iPS.

iPS cells cultured in differentiation medium containing 0.2% (*w*/*v*) DMSO (Control) for 6 days were compared to undifferentiated iPS cells to evaluate changes in gene expression, using the TaqMan™ hPSC Scorecard™ Panel (Figure 3).

Self-replicating genes and mesoderm-related genes were evaluated comprehensively. When the gene expression level in iPS cells was set to 1, the relative gene expression level in differentiated cells decreased to 0.24-fold for self-replicating genes and increased to 3.56-fold for mesoderm-related genes. Endoderm-related gene expression increased 1.57-fold, and ectoderm-related gene expression increased 1.71-fold, indicating that differentiation into early trichoderm had progressed at the gene expression level.

### 3.3. Determination of Test Substance Concentration

**Figure 4:**
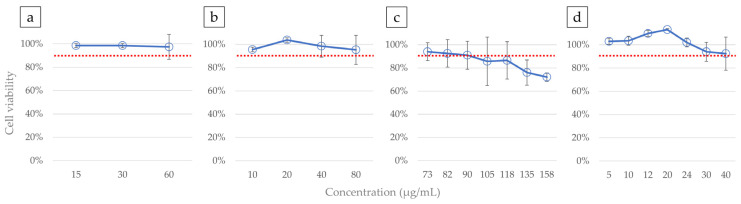
Percentage of cell viability at each concentration with the absorbance of the control WST-8 set to 100%: (**a**) Saxa, (**b**) Vilda, (**c**) VPA, and (**d**) Thalido. The red dotted line represents 90% cell viability (inhibitory concentration 10, IC10) relative to the control.

Dilution series were prepared based on the maximum solubility of each test substance, and cell viability was measured on day 6 of exposure. For Saxa, a dilution series was created from a 30 mg/mL stock solution in DMSO, which is near its maximum solubility, using medium containing 0.2% DMSO. Cell viability did not decrease to IC10, even at a final concentration of 60 µg/mL in the medium (Figure 4a). For Vilda, a dilution series was prepared from a 40 mg/mL stock solution in DMSO, also near its maximum solubility. Cell viability remained above IC10 at a final concentration of 80 µg/mL in the medium (Figure 4b). VPA was sourced as an ethanol solution; hence, a dilution series was prepared from a 100 mg/mL stock solution in EtOH using medium containing 0.5% DMSO. Exposure to a final concentration of 90 µg/mL reduced cell viability to IC10 (Figure 4c). For Thalido, a dilution series was prepared from a 20 mg/mL stock solution in DMSO, close to its maximum solubility in the medium. Cell viability did not decrease to IC10, even at a final concentration of 40 µg/mL in the medium (Figure 4d).

**Figure 5:**
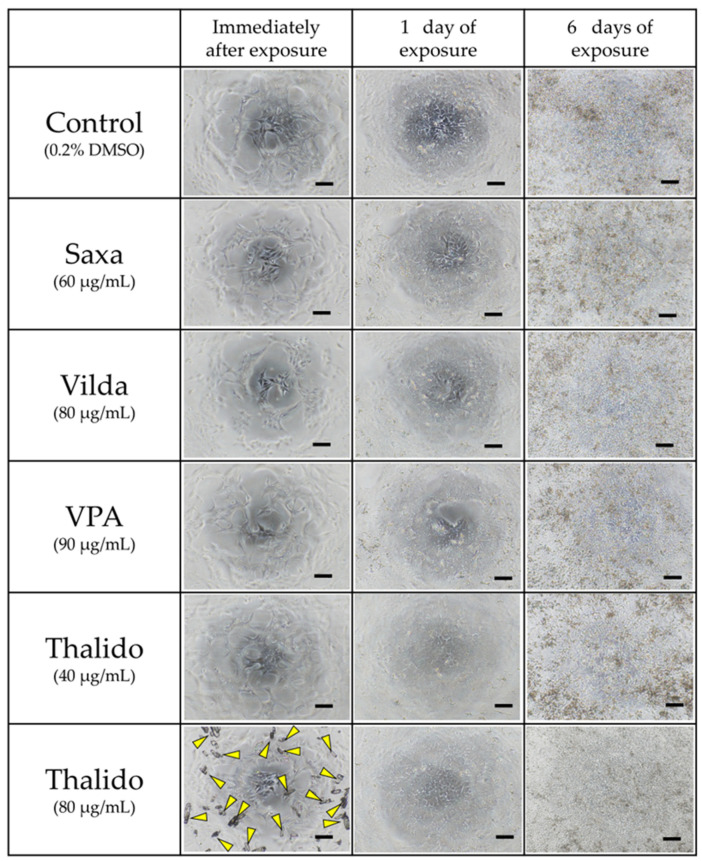
Condition of cells exposed at the applicable final concentration. Observations were made immediately after exposure (left column), 1 day after exposure (center column), and 6 days after exposure (right column). Yellow arrowheads indicate crystals precipitated when the Thalido stock solution (40 mg/mL in DMSO) was diluted in medium containing DMSO. Bar indicates 200 µm.

Cells were observed under an inverted microscope when exposed to the applicable final concentration (Figure 5). Although not mentioned in the package insert, Thalido could be dissolved at 40 mg/mL in DMSO. However, when diluted in medium containing 0.2% DMSO (final concentration: 80 µg/mL), crystals precipitated immediately. Therefore, a concentration of 40 µg/mL, prepared using a one-step dilution series, was used as the applicable final concentration. In the case of exposure at 80 µg/mL, Thalido crystals disappeared after 24 h of incubation.

The test substance application concentrations were determined based on the established criteria (Table 1), and the final concentrations for each test substance were defined in Table 3.

### 3.4. Analysis of RNA-Seq Results and Refinement of Candidate Genes

**Figure 6:**
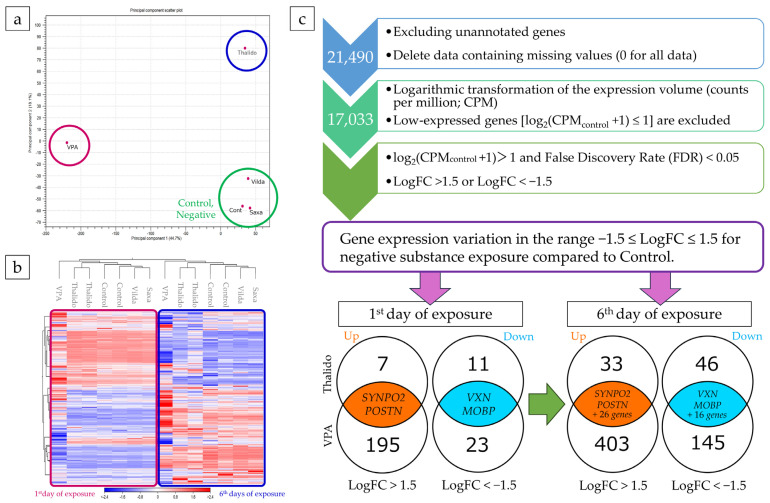
Results of raw data analysis obtained by RNA-seq. (**a**) PCA, (**b**) heatmap clustering, (**c**) and flowchart and Venn diagrams for candidate gene refinement. *SYNPO2*: *Synaptopodin 2*; *POSTN*: *Periostin*; *VXN*: *Vexin*; *MOBP*: *Myelin-associated oligodendrocyte basic protein*.

Raw RNA-seq data were analyzed. In the principal component analysis (PCA) performed on exposure day 1, the axis with the largest data variance was identified as the first principal component (PC1, 44.7%). The axis with next largest variance, orthogonal to the PC1, was identified as the second principal component (PC2, 18.1%). In the PCA plot of PC1 and PC2, control and negative substances (Saxa, Vilda) were positioned in a similar region. In contrast, VPA, used as a positive control in this study, showed distinct separation from controls and negatives along the PC1 axis, indicating its strong positive nature. Thalido, while plotted closer to the controls and negatives, showed some differences along PC1, suggesting it as a weakly positive substance (Figure 6a).

Heatmap clustering analysis results on days 1 and 6 of exposure revealed the following trends. For control, negative substances, and Thalido, gene expression followed similar patterns. Some genes were upregulated or downregulated with VPA exposure. On day 6, most genes upregulated on day 1 were downregulated in controls, negatives, and Thalido, and vice versa. VPA exhibited stronger changes in gene expression compared to controls, negatives, and Thalido (Figure 6b).

To narrow down candidate genes, comparisons were made by evaluating genes with minimal expression levels due to exposure to negative substances (Saxa and Vilda), changed expression due to positive control substance (VPA) and Thalido. First, unannotated genes were removed from the raw RNA-seq data, followed by the removal of data containing missing values (genes with zero expression in all samples). This process reduced the number of genes from 21,490 in the raw RNA-seq data to 17,033. Next, the expression levels (counts per million, CPM) were log-transformed [log_2_(CPM + 1)]. Genes with log_2_(CPM_control_ + 1) ≤ 1 (about 25% of the genes in the lower half of the expression) were excluded from the candidate genes as low-expression genes. Candidate genes that satisfied the conditions log_2_(CPM_control_ + 1) > 1, FDR < 0.05, and LogFC > 1.5 or LogFC < −1.5 were selected on exposure days 1 and 6. The resulting Venn diagram is shown in Figure 6c. Genes with increased or decreased expression in both or either of VPA and Thalido were further selected on day 6 as on day 1. These genes were subjected to GO analysis using DAVID Bioinformatics, and genes classified as being involved in developmental processes (*p*-value = 2.95 × 10^−5^ adjusted *p*-value; Benjamini–Hochberg method = 5.02 × 10^−4^) and in the biological processes were selected as candidate genes. The seven candidate genes were *SYNPO2*, *POSTIN*, *ROPN1*, *TP63*, *HPGD*, *ALPK2*, and *LTA*.

### 3.5. Confirmatory Analysis of Gene Expression Levels by qPCR

**Figure 7:**
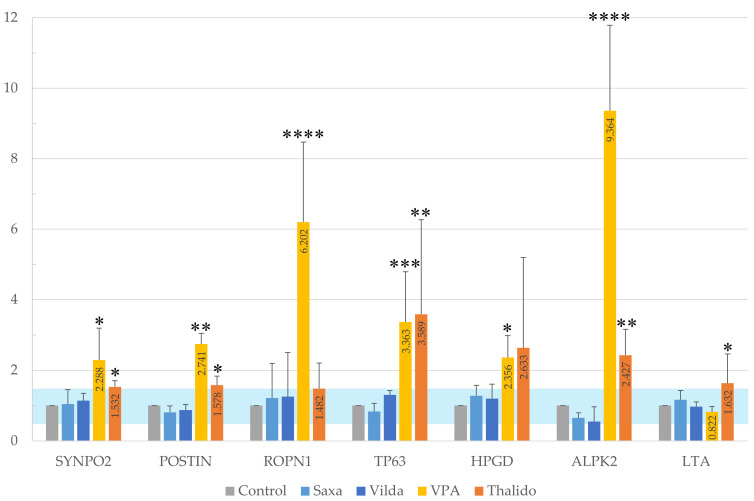
Results of confirmatory analysis of relative gene expression levels by qPCR for seven candidate genes. The blue region indicates a range of −1.5 ≤ LogFC ≤ 1.5 compared to the control. Significant differences: * *p* < 0.05; ** *p* < 0.01; *** *p* < 0.005; **** *p* < 0.001.

The expression levels of the seven genes were reanalyzed in triplicate using qPCR. The results indicated that the relative expression levels of the genes ranged from 0.5- to 1.5- fold when exposed to the negative substances Saxa and Vilda, with the control serving as the reference (Table 1). In contrast, genes with relative expression levels of ≥1.5-fold for both as a positive control of VPA and Thalido compared to the controls were SYNPO2, POSTIN, TP63, HPGD, and ALPK2. ROPN1 showed a ≥1.5-fold relative expression only for VPA, while LTA showed a ≥1.5-fold relative expression only for Thalido (Figure 7).

## 4. Discussion

In this study, we established a protocol for test substance exposure using human iPS cells and developed a novel method for detecting Thalido. By comprehensively analyzing RNA expression following exposure to VPA or Thalido, we identified seven candidate genes whose expression levels were altered by VPA and/or Thalido.

When reprogrammed iPS cells differentiate, epigenetic memory facilitates their differentiation into cells of origin [13]. In this study, iPS cells derived from human vascular endothelial cells were used, and the 6-day differentiation culture yielded a high mesoderm score among the three germ layers. However, genetic changes in the ectoderm and endoderm were only slightly increased compared to iPS cells, suggesting that the 6-day protocol differentiated the cells into a relatively early trichoderm state. With this protocol, cells became over-confluent after 6 days of culture, making it difficult to continue the culture for additional days. The gestational period, during which teratogenicity is most problematic, is considered to be from 4 to 7 weeks [14] and extends until approximately 15 weeks. Therefore, it is necessary to develop a culture method (e.g., spheroid culture, organoid culture) that supports the observation of differentiated cells over a longer duration. Additionally, depending on the number of days of differentiation culture, the expression balance of the trichoderm may vary [12]. It is expected that the iPS cells used in this study could exhibit higher levels of gene expression in ectodermal and endodermal lineages at specific times based on the culture duration. Pluripotent stem cell-based model assays have potential applications in the field of alternative methods for developmental toxicity testing [15].

FBS has been widely used as a differentiation induction medium in many studies. However, the variability in differentiation factors between lots can significantly impact sensitive gene expression analysis experiments. To address this issue, we successfully developed a differentiation medium based on a fully synthetic formulation. By incorporating factors that strongly induce differentiation into the trichoderm, we believe this medium can serve as a more specific differentiation induction medium. The criteria established in this study should be regarded as preliminary indicators, which we anticipate will be refined through further research in the future.

In this study, Thalido did not affect cell viability even at its maximum dissolved concentration. However, changes in gene expression related to development were observed, indicating that developmental toxicity, such as teratogenicity, can occur independently of cytotoxicity. When exposed to 80 μg/mL, which exceeded the maximum dissolution concentration, thalidomide crystals formed but dissolved within 24 h, likely due to the effects of temperature, pH, and components of the differentiation medium. Since these crystals could influence cell behavior as external factors and create differing exposure conditions, the Thalido concentration in this study was set at 40 µg/mL.

In this assay, substances that increase or alter gene expression between day 1 and day 6 of exposure can be evaluated as posing an increased risk with continued exposure. By examining a wide range of positive substances, we aim to clarify the relationships among factors such as AUC, Cmax, Tmax, absorption rate constant, elimination rate constant, ionization, fat solubility, concentration gradient, molecular weight, and protein binding rate. Additionally, the impact of changes in a pregnant woman’s internal environment on pharmacokinetics, such as increased blood volume and drug passage through the placenta, requires further investigation [16]. Drugs that readily cross the placenta often exhibit specific characteristics: small molecular weight (M.W. less than 1000), lipophilicity (free passage), non-ionized form (given that fetal blood pH is slightly lower than maternal), and the presence of transporters such as P-glycoprotein, which facilitates the transport of lipidic substances) [17]. The condition of the placenta, including its growth with advancing gestational age and the development of the fetal–maternal blood barrier, as well as drug metabolism capacity (involving CYP3A4 and CYP2C enzyme groups), may also influence drug passage [18]. When a pregnant woman takes a drug, it is absorbed in the maternal intestinal tract, enters the bloodstream, binds partially to albumin, and undergoes metabolism in the liver through cytochrome P450 enzymes and glucuronidation. The drug then crosses the placenta and reaches the fetus as umbilical cord blood. Therefore, understanding the metabolism of substances, including drugs, is essential for evaluating reproductive and developmental toxicity.

The positive control for this study of VPA, including its derivative sodium valproate, is commonly used to treat bipolar disorder and as an antiepileptic drug, but it is contraindicated during pregnancy due to risk of neural tube defects, morphological birth defects, cognitive dysfunction, and developmental disorders in the fetus [19,20,21]. The teratogenic effects of VPA have been reported in mice, with detailed studies also observing teratogenicity in VPA isomers, such as the amide derivative valnoctamide [22]. Additionally, VPA interferes with the activity of gene-regulating enzymes, specifically histone deacetylases. Since histone deacetylases remain active during the fetal period, congenital malformations, and higher brain dysfunction caused by VPA exposure may result from its inhibitory effect on these enzymes [23]. Similarly to this study, a genome-wide expression screening study of VPA exposure in zebrafish embryos reported 62 genes as biomarkers of early neurodevelopmental toxicity [24].

In 2010, the protein cereblon was identified as a major intracellular target of thalidomide, associated with limb and ear deformities [25]. Further research revealed that a neo-substrate involved in thalidomide’s teratogenicity is a protein called p63. Studies using a zebrafish model revealed that thalidomide inhibits limb and ear development by inducing the degradation of p63 [26], which is a protein encoded by TP63 (tumor protein p63) gene in humans [27,28,29,30]. As a transcription factor within the p53 family, p63 plays an important role in development. Mice deficient in p63 exhibit multiple developmental abnormalities, including limb defects and defects in tissues such as teeth and mammary glands, where development occurs through mesenchyme–epithelium interactions [31]. In this study, TP63 was also selected as a candidate gene. Comprehensive RNA-seq analysis of the differentiation stage to early trichoderm revealed relationships between thalidomide, control substances, and negative substances. These findings may provide insights into factors unique to thalidomide detection, particularly its species-specific characteristics. We believe this approach could aid in elucidating the mechanisms underlying thalidomide’s detection and species-specificity. Mutations in TP63 have been reported to cause at least six different syndromes, in which ectodermal dysplasia, cleft lip, and limb malformations appear in various combinations [32]. However, the pattern of limb abnormalities associated with this TP63 mutation is markedly different from that seen in thalidomide embryopathy.

Six candidate genes in addition to *TP63*, were identified from GeneCards^®^, the human gene database [33]. (1) *SYNPO2* (*Synaptopodin 2*)—This gene is associated with α-actinin and filamin binding activity. It plays a role in regulating cell migration. (2) *POSTIN* (*Periostin*)—This gene encodes an extracellular matrix protein that promotes cell adhesion and spreading. It is involved in tissue development and regeneration, including wound healing. (3) *ROPN1* (*Rhophilin Associated Tail Protein 1*)—This gene contributes to the integrity of the sperm fiber sheath, regulates sperm motility, and is essential for PKA-dependent signaling processes required for fertilization. (4) *ROPN2* (*Rhophilin Associated Tail Protein 2*)—This encodes a protein involved in regulating sperm motility. (5) *ALPK2* (*Alpha Kinase 2*)—This gene encodes a protein kinase that targets phosphorylation sites in peptides with an alpha helix structure. (6) *LTA* (*Lymphotoxin Alpha*)—This is a cytokine that, in its homomeric form, binds to TNFRSF1A/TNFR1, TNFRSF1B/TNFBR, and TNFRSF14/HVEM. It plays a role in the formation of secondary lymphoid organs during development and in apoptosis. Each of these genes is associated with tissue development and cell growth, with their respective functions contributing to developmental processes or physiological regulation. In a previously published paper using zebrafish, among the seven candidate genes detected in this study, in addition to the aforementioned *TP63*, *SYNPO2* was reported to be associated with congenital muscle disease [34] and *ALPK2* has been implicated in heart formation [35,36].

At this stage, the candidate genes identified remain provisional, and it is unclear whether they may fully account for the detection of all ICH S5(R3) substances in further validation studies. However, using the experimental methods applied in this study, we plan to categorize ICH S5(R3) substances based on their properties and revalidate them through RNA-seq analysis. Specifically, the classification of the physical properties of emerging modality drugs and chemicals, such as nucleic acid-based drugs and antibody drugs, according to the human genome is possible with current science and technology. We aim to construct an evaluation system incorporating multiple candidate genes to assess developmental toxicity based on a physical property classification of test substances.

Compared to traditional animal experiments, this method offers advantages in terms of time and labor efficiency and, most importantly, in terms of animal welfare. However, cell experiments may not be able to fully evaluate cell–cell interactions, endocrine effects, and the metabolism of the test substance as effectively as in vivo experiments. As an alternative to phylogenetically lower animals, zebrafish have been used in non-mammalian animal tests. It was reported that when zebrafish were exposed to Thalido, abnormalities in pectoral fin and otocyst development were observed due to the degradation of Thalido’s target factor, Cereblon, into delta-Np63α and Tap63α [26]. Additionally, the organoid culture model, another cell-based test method, has gained attention as a complementary approach to two-dimensional cell cultures, with the organoid models of various tissues being reported [37,38].

This study has some limitations. Only two CH S5(R3) positive substances, VPA and Thalido, were validated. Additionally, the assay evaluated the time period from the fertilized egg stage to relatively early trichoderm differentiation. Among the ICH S5(R3) substances, VPA and Thalido are relatively soluble, while insoluble substances were not examined. Nonetheless, we believe that expanding this assay to include diverse culture and exposure conditions, combined with AI-driven analysis, could uncover genetic variations influenced by factors such as polarity and pH and support the detection of currently unknown substances.

## 5. Conclusions

This study established a protocol to evaluate the effects test substance exposure during the differentiation of human iPS cells without FBS, using cellular activity and genetic analysis. This protocol successfully detected Thalido, and comprehensive RNA expression analysis identified up to seven candidate genes with altered expression levels following exposure to VPA or Thalido. The refinement of this assay hold promise for developing new alternative methods for developmental toxicity testing using human iPS cells.

## 6. Patents

The contents of this manuscript are the subject of a pending patent in Japan (2024-191215).

## Figures and Tables

**Table 1:** Criteria to establish applicable concentrations of test substances.

To minimize any influence of the solvent (DMSO), the test substance stock solution was diluted 500-fold with medium.
The upper concentration limit was the maximum dissolved concentration of the test substance.
The lower concentration limit was derived from the blood concentration area under the curve (AUC) described in the test substance’s package insert.
The concentration was adjusted to prevent crystals precipitation when diluted in culture medium.
The IC_10_ (inhibitory concentration 10) was defined as the concentration reducing cell viability by approximately 10% compared to the control after 6 days of exposure.

**Table 2:** Genes analyzed by qRT-PCR and their TaqMan™ Primer & Probe sets.

Gene Name	Gene Symbol	Assay ID
*Synaptopodin 2*	*SYNPO2*	Hs00326493_m1
*Periostin*	*POSTIN*	Hs01566750_m1
*Rhophilin Associated Tail Protein 1*	*ROPN1*	Hs00375051_m1
*Tumor Protein p63*	*TP63*	Hs00978340_m1
*Hydroxyprostaglandin Dehydrogenase*	*HPGD*	Hs00960590_m1
*Alpha Kinase 2*	*ALPK2*	Hs01085415_g1
*Lymphotoxin alpha*	*LTA*	Hs06633590_s1
*Actin Beta*	*ACTB*	Hs99999903_m1

**Table 3:** CAS Registry Number^®^ (Cas RN^®^), ICH S5(R3) final applicable concentration, interview form (standard for minimum added concentration), maximum dissolved concentration based on the package insert, and final applicable concentration of each test substance.

	Test Substance	CAS RN^®^	ICH S5(R3) ^1^ (Cmax, AUC)	Interview Form (Cmax)	Package Insert (Maximum Dissolved Concentration ^2^)	Final Applicable Concentration
Negative at ICH S5(R3)	Saxa	361442-04-8	0.024 µg/mL, 0.078 µg·h/mL	0.05 µg/mL	34 mg/mL	60 µg/mL
	Vilda	274901-16-5	N/A, 2.06 µg·h/mL	0.6 µg/mL	45 mg/mL	80 µg/mL
Positive at ICH S5(R3)	VPA	99-66-1	205 µg/mL, 4180 µg·h/mL	120 µg/mL	100 mg/mL	90 µg/mL
	Thalido	50-35-1	0.62 µg/mL, 4.9 µg·h/mL	3 µg/mL	20 mg/mL	40 µg/mL

^1^ ICH-S5(R3) [1], ^2^ Package inserts.

## Data Availability

Dataset available on request from the authors.

## Supplementary Materials

The following are available online at https://www.mdpi.com/article/10.3390/cells14030215/s1: Supplementary Figure S1: Comparison of cell viability in different compositions of differentiation medium. a: 5%FBS with DMEM; b: 5%KSR with MEM NEAA, Gluta MAX, and PC/SM in Human Plasma-like Medium; c: 5%KSR with MEM NEAA, Gluta MAX, and PC/SM in DMEM; d: 5%KSR, with MEM NEAA, Gluta MAX, ITS-X, Monothioglycerol, and PC/SM in DMEM. Supplementary Figure S2: DMSO concentration in relation to the addition of the test substance. The WST-8 test showed no difference in cell viability on days 2, 4, and 6 of culture at different concentrations of DMSO in the control. Supplementary Table S1: Positive and negative substances in the qualification of alternative methods for ICH-S5(R3). Supplementary Figure S3: Expression level of β-actin as endogenous control. Trichoderm differentiation and exposure to the test substance did not alter the expression level of β-actin in the endogeneous control.
