# Supplementary figures and images for: Comprehensive Search for Genes Involved in Thalidomide Teratogenicity Using Early Differentiation Models of Human Induced Pluripotent Stem Cells: Potential Applications in Reproductive and Developmental Toxicity Testing
Source: Cells. 2025 Feb 2;14(3):215. doi: 10.3390/cells14030215 (PMC11817626; doi:10.3390/cells14030215)

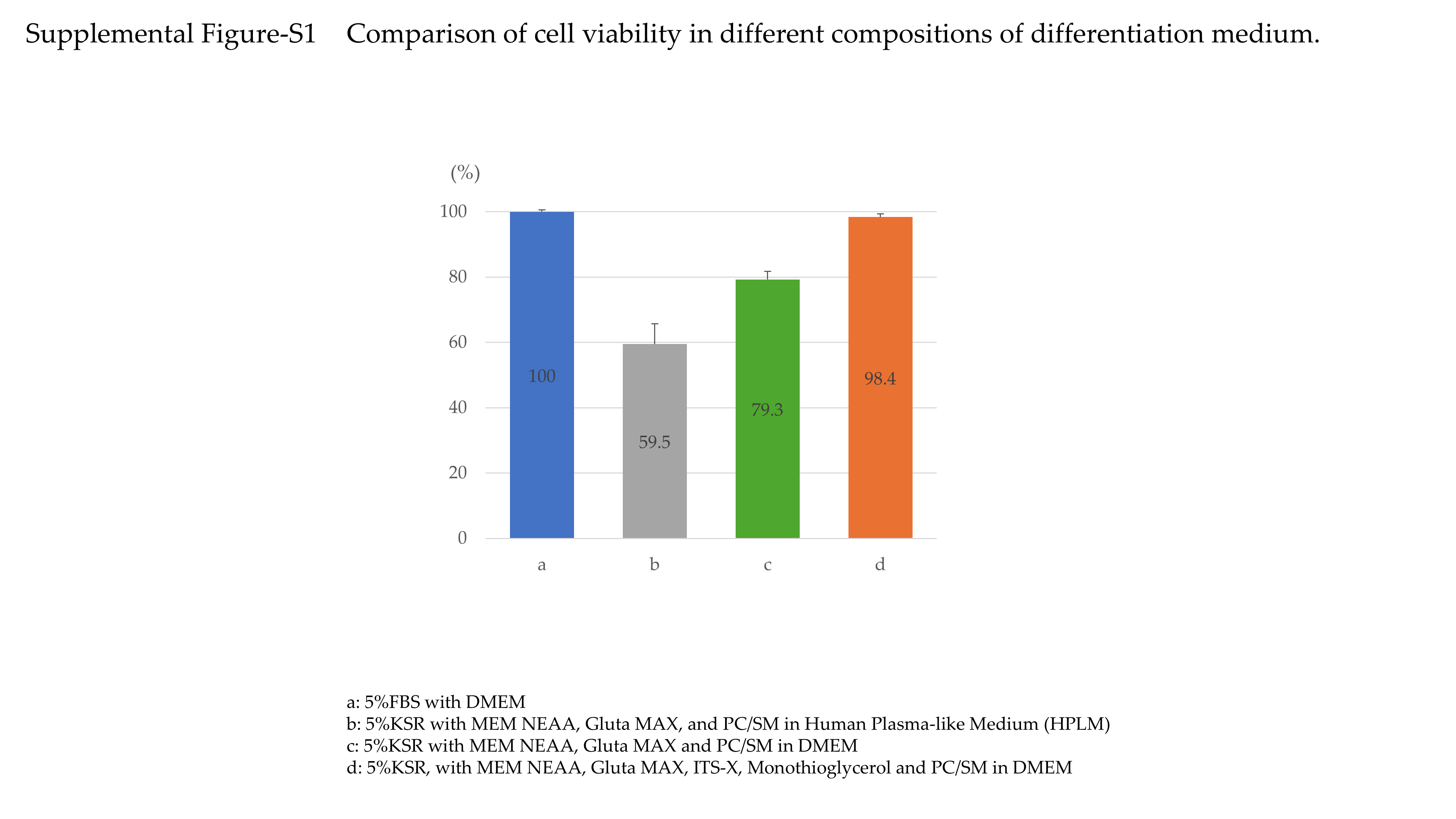

Supplement: Supplementary file 1 [file cells-14-00215-s001.zip › Supplemental Figure-S1.TIF]

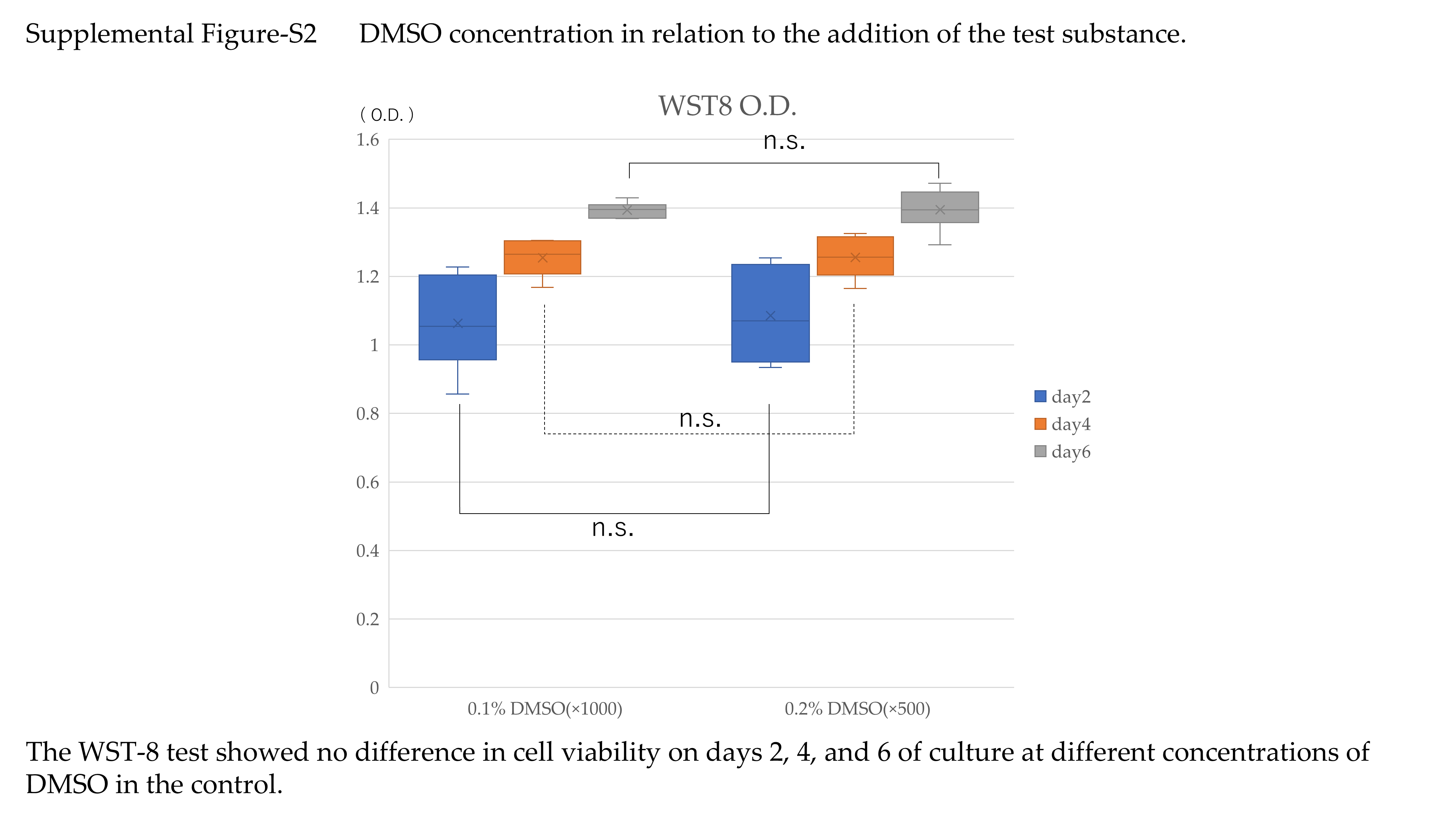

Supplement: Supplementary file 1 [file cells-14-00215-s001.zip › Supplemental Figure-S2.TIF]

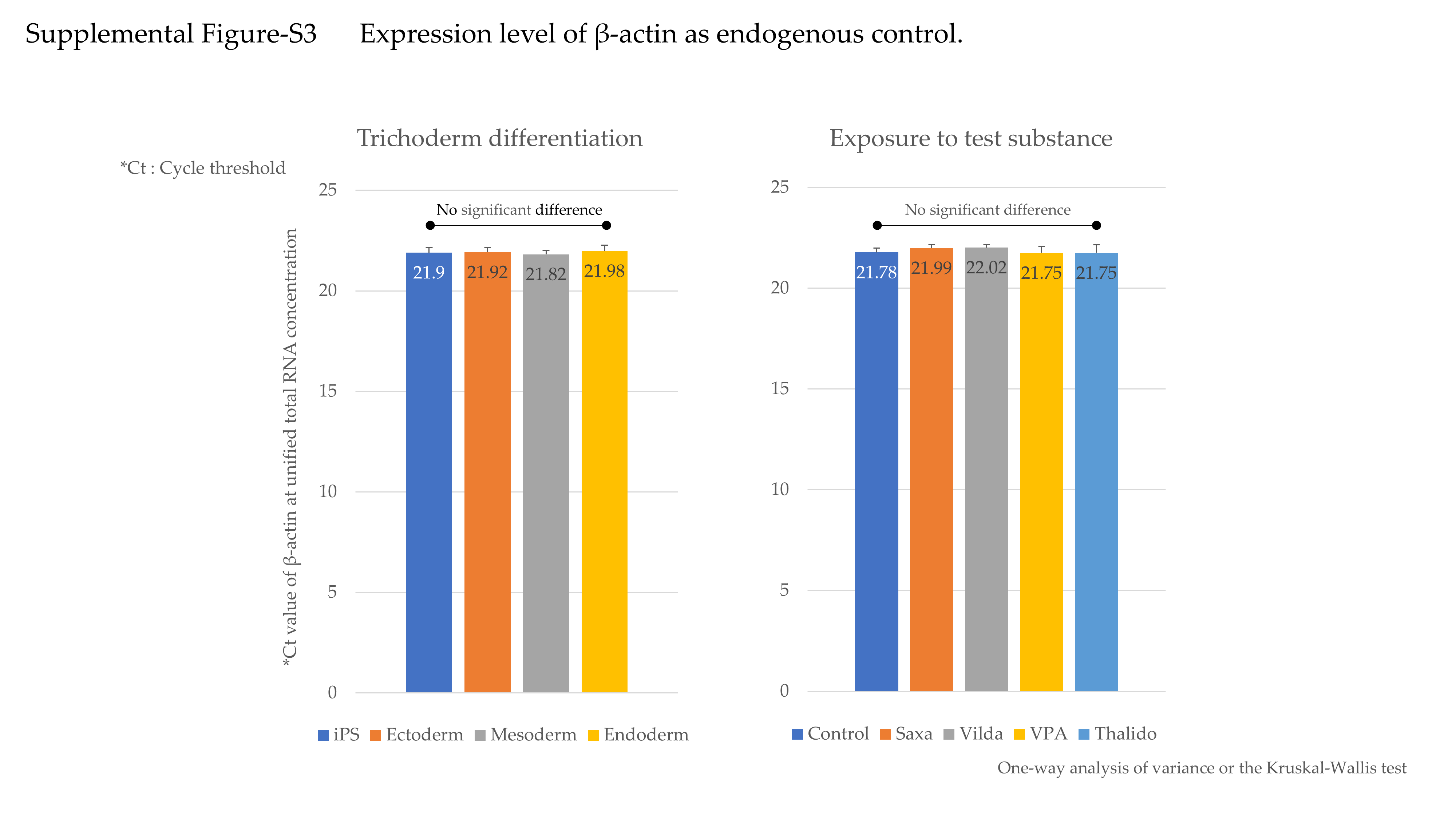

Supplement: Supplementary file 1 [file cells-14-00215-s001.zip › Supplemental Figure-S3.TIF]

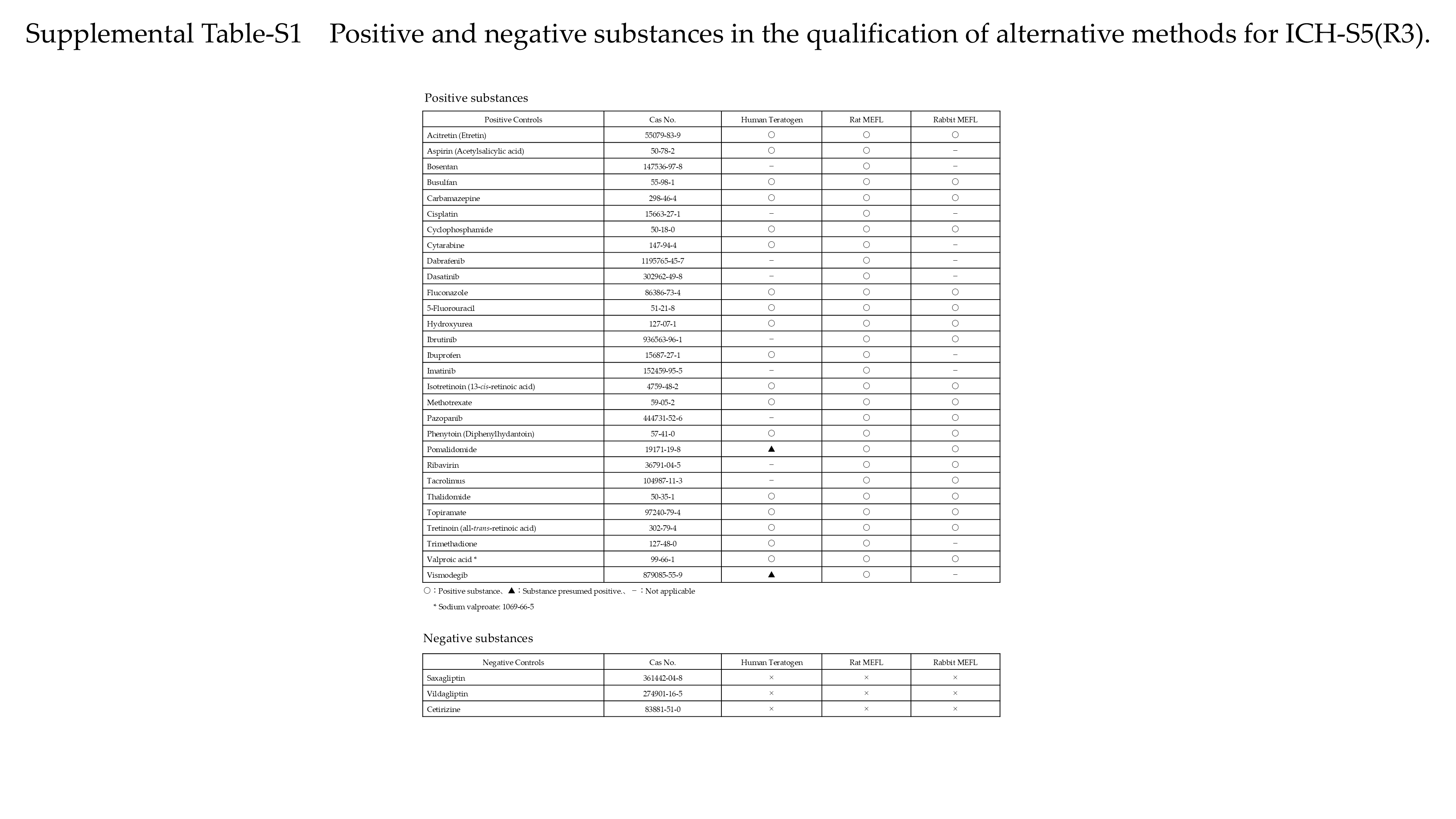

Supplement: Supplementary file 1 [file cells-14-00215-s001.zip › Supplemental Table-S1.TIF]
